# Radio-Telemetric Assessment of Cardiac Variables and Locomotion With Experimentally Induced Hypermagnesemia in Horses Using Chronically Implanted Catheters

**DOI:** 10.3389/fvets.2019.00414

**Published:** 2019-11-21

**Authors:** Stephen A. Schumacher, Ramiro E. Toribio, Jeffrey Lakritz, Alicia L. Bertone

**Affiliations:** ^1^Department of Veterinary Clinical Sciences, College of Veterinary Medicine, The Ohio State University, Columbus, OH, United States; ^2^The United States Equestrian Federation, Equine Drugs and Medications Program, Columbus, OH, United States

**Keywords:** magnesium sulfate, Mg^2+^, Ca^2+^, Ca^2+^/Mg^2+^, telemetry

## Abstract

The objective of this study was to characterize the pharmacokinetics and pharmacodynamics of intravenous administration of magnesium sulfate to horses using a novel radio-telemetry system for physiologic signal capture. Five Horses were surgically implanted with a radio-telemetric carotid catheter. Implants were paired with a non-invasive telemetric unit which acquired a six lead ECG and 3-axis acceleration to assess activity acquired wirelessly in real-time for future analysis. Horses were exposed to a new stall environment before (baseline) and after 60 mg/kg (30 mL) of magnesium sulfate (MgSO_4_), or the same volume of 0.9% saline, administered intravenously in a blinded, random crossover design. Blood for pharmacokinetics, telemetric data, and body temperature were recorded serially for 24 h. Data were analyzed across time and between treatments. Significance was set at *P* < 0.05. Ionized magnesium concentration (Mg^2+^) increased and the Ca^2+^ to Mg^2+^ ratio decreased and persisted for 5 h after MgSO_4_ administration. Heart rate (HR) increased and mean arterial blood pressure (MAP) decreased for at least 6 h. Electrocardiogram (ECG) intervals (RR) decreased and (PR and QTc) increased in duration compared to controls indicating an increase in heart rate, and slower myocardial conduction in the MgSO_4_ group. Acceleration in all planes was less in the MgSO_4_ group compared to controls indicating decreased locomotion. This novel method permitted collection of physiologic signals without interference by handlers or animal restraint. An intravenous bolus of MgSO_4_ produced cardiac variable changes associated with the reduction of locomotion in these horses, and in a direction that may be causal. Locomotion was decreased when horses were first introduced into a new environment which reflects the calming effect desired in sport horses. Telemetric monitoring can be used as a model to elucidate the behavior and physiologic effects of other drugs. The administration of MgSO_4_ may be detected for regulatory purposes with the monitoring of Mg^2+^ and Ca^2+^ concentrations and their ratio.

## Introduction

The abuse of magnesium sulfate (MgSO_4_) is a regulatory issue in equestrian sport as it is used to calm horses in competition (1). The Federation Equestre Internationale (FEI), the international governing body of equestrian sport, has made the administration of MgSO_4_ a prohibited practice and has listed MgSO_4_ on the Equine Prohibited Substances List (EPSL)^1^ due to its potential for calming and abuse, but evidence is predominantly anecdotal. The United States Equestrian Federations (USEF) Equine Drugs and Medications Rules prohibit the use of injections within the 12 h prior to competition, but at this time does not prohibit MgSO_4_. The USEF is the recognized national governing body of equestrian sport. If evidence is identified that the administration of MgSO_4_ is a behavior modifying substance, USEF will likely reevaluate its stance on the substance. However, the detection of MgSO_4_ administration is difficult because the endogenous nature of magnesium and its active form, ionized magnesium (Mg^2+^). To introduce regulatory control requires a method to differentiate between normal Mg^2+^ and related electrolyte concentrations and changes in these concentrations because of MgSO_4_ administration. Magnesium is very important for countless physiologic functions and one of the most abundant elements in all mammals. Magnesium is considered a calcium channel blocker, both centrally blocking Ca^2+^ at NMDA receptors in the CNS and in peripheral vessels (2). MgSO_4_ is commonly used in humans for the treatment of preeclampsia in women (3–5), post stroke prevention of hypoxia induced glutamate excitotoxicity (6), the perioperative management of pheochromocytomas (7), and the treatment of acute asthma (8–10). In horses, MgSO_4_ is used to treat large colon impactions (11, 12) and there has been recent investigation into its use for the treatment of trigeminal neuritis (13).

In previous work, a decrease in mean arterial pressure (MAP) from baseline was detected immediately following the administration of MgSO_4_; concurrently, there was an increase in heart rate (Schumacher et al., submitted). The horses included in previous work were confined in stocks for 6 h for the duration of the experiment. There was no control group due to the difficulty with restraining the horses, as instrumented, for the 6 h without the administration of MgSO_4_. It is difficult to assess the pharmacodynamic effects of drugs on animals when restraint of the animal is required. To address these limitations and prove a physiologic effect on blood pressure and heart rate as well as behavior calming, a telemetric assessment of these parameters to study horses in a free and natural environment was necessary.

The goals of this experiment were to evaluate the effects of intravenous MgSO_4_ on arterial pressure, identify changes in the electrocardiogram, and locomotion in unrestrained horses. Our hypotheses were that the pharmacokinetics and decrease in blood pressure, compared to the 0.9% NaCl control, would be similar to our prior work; and utilizing sensitive accelerometers, a decrease in locomotion would be detected in the MgSO_4_ treatment group compared to controls. We will also confirm that plasma subjected to the standard collection, storage, and shipping methods of the United States Equestrian Federation's Equine Drugs and Medications Program will have similar Mg^2+^ and Ca^2+^ as previously published (14).

## Materials and Methods

### Animals

Five healthy adult (median age 8 years [range, 4–9 years]) university-owned Quarter Horse (*n* = 4) and Standardbred (*n* = 1) mares with a mean weight of 1,247 lbs were included in the study. This study was approved by the University Institutional Animal Care and Use Committee (IACUC) and fulfilled ARRIVE guidelines (https://www.nc3rs.org.uk/arrive-guidelines) for the humane use of animals in research. All horses were deemed healthy following physical exam. All horses were vaccinated and dewormed at least 1 month prior to inclusion. Housing for the duration of the study was in the Ohio State University Medical Center, in box stalls (3.6 × 3.6 m), and horses were fed grass hay and water *ad libitum*.

### Study Design

This study was a blinded, crossover study with each horse receiving a 60 mg/kg intravenous administration of MgSO_4_, and an equivalent volume dose of 0.9% NaCl. A minimum 1-week washout period was observed between randomized treatments. All administrations were infused over 5 min. Physiologic signals and plasma samples were collected serially (5, 15, 30 min, and at 1, 2, 3, 4, 5, 6, 12, and 24 h).

### Surgical Implantation of Arterial Catheter

Implantation was conducted at least 2 weeks prior to experimental data collection to allow time for recovery from the implantation procedure. Horses were premedicated with 1.1 mg/kg of xylazine (AnaSed® Akorn, Lake Forest, IL), and then placed under general anesthesia using a mixture of 2.2 mg/kg ketamine (VetaKet® Akorn, Lake Forest, IL) and 0.06 mg/kg of midazolam (Novaplus® West-Ward, Eatontown, NJ). Horses were intubated and maintained under general anesthesia using isoflurane (Akorn, Lake Forest, IL). During the surgical procedure, heart rate, respiration rate, electrocardiogram and depth of anesthesia were monitored to ensure appropriate plane of anesthesia. Horses were placed in right lateral recumbency with limbs secured. Following appropriate sterile technique to prepare the surgical site, a skin incision (~4in) was made in the caudal-neck longitudinally along the jugular furrow. The left carotid artery was identified and exteriorized with umbilical tape securing the vessel above the plane of the skin. A purse string suture pattern was preplaced around the site identified for catheter insertion using 5-0 non-absorbable nylon suture. A #11 scalpel blade was used to create a small stab incision in the carotid artery, and a 16 g gel filled 35 cm long arterial telemetric catheter (easyTEL +_L_PT g35, Emka Technologies, Falls Church, VA) was threaded into the carotid artery and advanced toward the subclavian artery. Approximately 8 cm remained outside of the vessel. The catheter was secured with a tightening of the preplaced purse-string suture. The transducer/battery pack were secured in a subcutaneous pocket of muscle and subcutaneous tissue lateral to by 2-0 non-absorbable nylon suture. Implant functionality was confirmed by recovery of arterial pressure waveforms. The subcutaneous fascia was closed using a continuous pattern and 2-0 absorbable suture. The skin was closed with 0 non-absorbable suture in a simple interrupted pattern. Horses were recovered from general anesthesia and continuously monitored until they could stand. Implant functionality was again confirmed and horses were observed every 6 h for the following 48 h. Phenylbutazone (Vetone®, Boise, ID) was administered intravenously once daily for 4 days at a dose of 4.4 mg/kg, and procaine penicillin (PenOne Pro™;Vetone®, Boise, ID) was administered intramuscularly for 4 days at a dose 6,600 units/kg. Skin sutures were removed on day 14 post surgery. Horses were not included in this study until at least 28 days post-implantation surgery.

### External Jugular Catheter Placement

On the day of the experiment, two 14-guage catheters were placed aseptically; one in each external jugular vein. All placement of jugular catheters took place in the horse's regular stall and not in the treatment stall. The right jugular catheter was used solely for the administration of MgSO_4_, and the left jugular catheter for blood collection. The right jugular catheter was removed after administration of the total dose of MgSO_4_ or 0.9% NaCl.

### Instrumentation for Radio-Telemetric Data Collection

**Figure 1:**
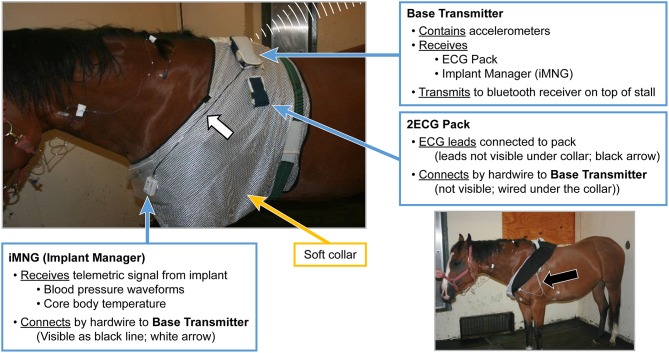
Picture of horse with soft collar applied and the connections of iMNG (Implant Manager), and the 2ECG Pack, to the Base Transmitter. Black Arrow identifying ECG leads and electrode placement.

**Figure 2:**
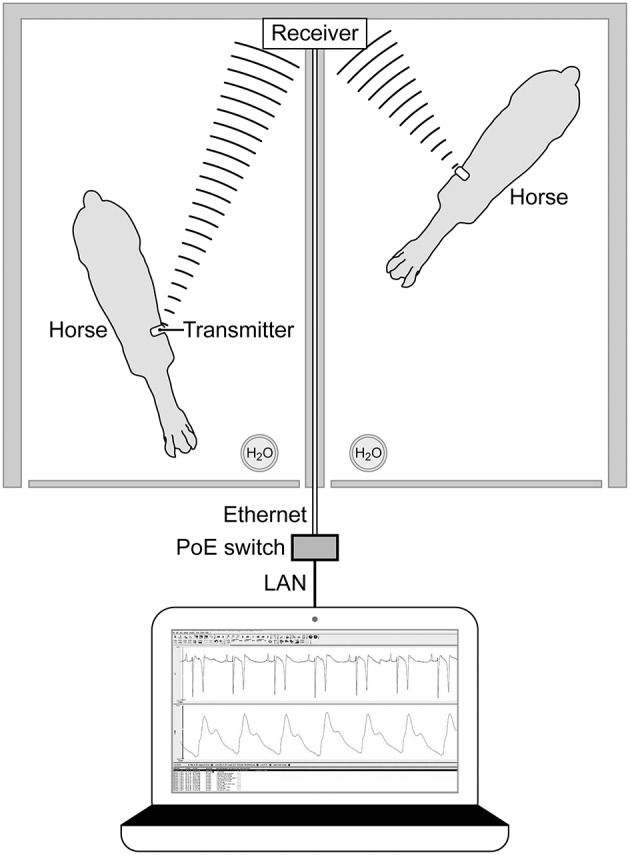
Overhead diagram horses and Bluetooth receiver mounted on the top of the stall wall with Ethernet connection to computer for acquisition of signals. Bluetooth receiver collects signals from the base pack wireless transmitter on the withers of each horse.

**Figure 3:**
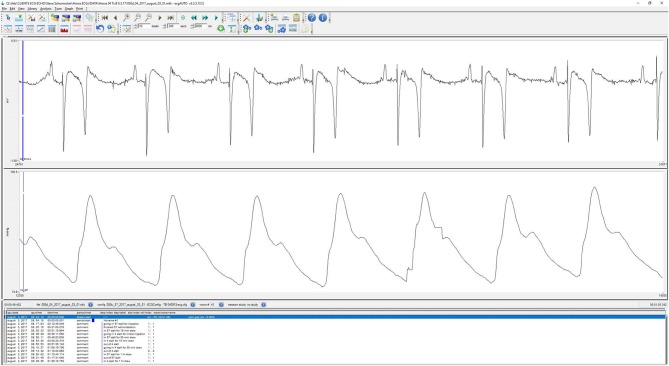
Characteristic screen shot of ECG and arterial blood pressure waveform during wireless Bluetooth acquisition from a horse.

On the day of the experiment, following the placement of the jugular catheters, horses were placed in a novel stall used for the experiment. Surface leads were attached to the horse using foam monitoring ECG electrodes (3M Maplewood, MN), and placed with the (green; RL) caudal to the olecranon, the (red; LL) superior to the (green) lead. The (white; RA) lead was placed along the cranial sternum with the neutral (black; LL) lead placed at the point of the shoulder. The surface leads for the ECG were directly connected to the (emkaPACK_4G_TR+_2ECG, EMKA Technologies, Falls Church, VA), which was connected via cable to the base transmitter (emkaPACK_4G_TR_base, EMKA Technologies, Falls Church, VA). The telemetric catheters, which were in dormant mode, were activated by placing a magnet over the subcutaneously placed transducer/battery pack. Battery life for catheter and transducer is ~200 h, which allows for numerous experiments if the catheter is turned off in between treatments/experiments. A lightweight, spandex collar was placed on the horses. The collar had been placed on each of the horses for an hour a day for 5 days the week prior to get them familiar with the process, no horses exhibited unusual behavior following conditioning with the spandex collar. This collar had pockets designed to hold the modules used for signal acquisition and relaying telemetric readings (Figure 1). The implant MANAGER module (emkaPACK_4G_TR_iMNG, EMKA Technologies, Falls Church, VA), which received signals from the implant was located at the point of the shoulder in close proximity to the subcutaneous transducer/battery pack. The signal from the implanted telemetric catheter and transducer was radio-transmitted to implant MANAGER module which was connected by a hard-wire to the base transmitter. The base transmitter was located in a pocket on the withers, and also housed the accelerometers responsible for monitoring activity. The electrocardiograms were obtained by surface electrodes placed in a base apex configuration. The base transmitter connected, via unique frequency, to the Bluetooth receiver (emkaPACK_4G_RE_16 Receiver, EMKA Technologies, Falls Church, VA). The Bluetooth receiver was capable of collecting unique signals from up to 12 base transmitters, but in our experiment, the receiver collected the signals from two horses on each day of the experiment. The Bluetooth receiver was attached to the top of the stall wall and was connected via Ethernet cable to the acquisition computer (Figure 2). Once the horses were properly instrumented, baseline readings were taken (Figure 3).

### Sample Collection

Horses were placed in a new stall for the first time ~30 min prior to the experiment, and baseline physiologic signals and plasma samples were collected. All experiments were conducted at the same time each day. The stalls were not bedded and water was *ad libitum* but no hay was provided during the first 6 h of continuous monitoring. Following MgSO_4_ or 0.9% NaCl (control) administration; both infused over 5 min, all physiologic signals and plasma samples were collected serially (5, 15, 30 min, and at 1, 2, 3, 4, 5, 6, 12, and 24 h). At each of the collection time points, blood pressure, ECG, and acceleration data were acquired for 2 min prior to study personnel entry into the stall for collection of plasma samples. With the exception of the time to collect plasma samples, horses were loose in the stall (3.6 × 3.6 meters). All interaction with the horses during the active experiment was limited to the collection of plasma samples from the jugular catheter.

#### Blood Collection

Plasma samples were collected anaerobically at each of the time points described above. A 10 cc syringe was used to collect waste blood to assure a fresh blood sample; the waste was discarded. Blood was collected using a 30 cc syringe and divided into two plasma separator tubes (8.0 ml PSTTM Gel and Lithium Heparin, BD Vacutainer, Franklin Lakes, NJ). The remaining blood was transferred to plain tubes and EDTA tubes; plain tubes were allowed to clot for 1 h, centrifuged at 1,000 g for 5 min at 4°C. Serum and plasma samples were aliquoted and stored at −80°C for later endocrine analysis.

#### Electrocardiogram

ECG signals were transmitted from the base to the Bluetooth receiver. Standard ECG intervals (RR, PR, QRS, and QT) were measured. The value for the QT interval was corrected for HR using Bazett's formula and provided a corrected QT interval (QTc). The monitor was evaluated by an observer to detect any failure to transmit or arrhythmias. Post experiment, ECG's were analyzed using ECG auto software package (ECG_AUTO_FULL, EMKA Technologies, Falls Church, VA).

#### Blood Pressure Measurements

All blood pressure measurements were acquired using the implanted telemetric catheters (easyTEL + implant). Real time arterial pressure waveforms were acquired and analyzed post-experiment for diastolic (DAP), systolic (SAP) and mean arterial pressures (MAP). Analysis was completed by a cardio analyzer software (ECG_AUTO_Cardio1+, EMKA Technologies, Falls Church, VA). Arterial waveforms were also analyzed for heart rate (HR), and +dP/dt (the maximum rate of rise in left ventricular pressure).

#### Temperature

The implanted blood pressure telemetric catheter, described above, also acquired core body temperature data and transmitted to the computer concurrently with the blood pressure data.

#### Locomotion

The base (emkaPACK_4G_TR_base) included accelerometers that measure in 3-axes (X, Y, and Z); the x-axis measured acceleration from side to side, the y-axis measured acceleration up and down, and the z-axis measured acceleration moving forward and backward. The overall measure of locomotion was taken as the square root of the sum of the squares for each of the 3-axes. This analysis was completed using the ECG_AUTO_Slow+ analyzer (EMKA Technologies, Falls Church, VA).

#### Observational Behavior Assessment

All horses were observed for head elevation, ear movement and overall movement. The same blinded observer made all behavior assessments of all horses. Head elevation was assessed on a scale range of 1–4; 1 was below the level of the neck, 2 was at the level of the neck, 3 was natural head position, and 4 was elevated head height. Ear movements were counted over 30 s at each time period, and movement was assessed as a binary function with ear movement given a score of 1 and ear non-movement given a score of 0.

## Data Analysis

### Blood

Plasma samples were refrigerated, then shipped by commercial shipper within 48 h to the United States Equestrian Federation's Equine Drug Testing and Research Laboratory (EDTRL) in Lexington, Kentucky to duplicate the process used in the collection and analysis of regulatory samples (14). A Nova pHox Ultra Analyzer (Nova Biomedical, Waltham, MA) was used to analyze plasma samples for pH, TCO_2_, Mg^2+^, and Ca^2+^, and provided the Ca^2+^/Mg^2+^ ratio.

### Post Experiment Review of Physiologic Data

All physiologic data (blood pressure, ECG, locomotion, and temperature) were analyzed by a contract research organization (QTest Labs, Columbus, OH) using ECG_AUTO_Cardio1+ cardio analyzer, ECG_AUTO_FULL, and ECG_AUTO_Slow+ software (EMKA Technologies, Falls Church, VA). For all objective measures, including blood pressure, heart rate, ECG intervals, and activity, data were recorded every 500 ms. Post study analysis consisted of averaging four consecutive 30 s intervals at each time point for a full 2 min of data.

### Pharmacokinetic Analysis

A commercially available pharmacokinetic software package was used to calculate the pharmacokinetic parameters (Phoenix WinNonlin, Version 8.0, Pharsight Corporation, Cary, NC, USA) using non-compartmental analysis. All plasma concentrations for Mg^2+^ were reported in mmol/L and the total dose administered was converted to mmol from milligrams elemental Mg^2+^. To normalize plasma concentrations, prior to pharmacokinetic analysis, the Mg^2+^ concentration from baseline (*T* = 0) was subtracted from each of the time points. The elimination rate constant (k_el_) was determined from the slope of the terminal portion of the plasma concentration × time curve. The terminal half-life (HL_Lamda_z) was determined by dividing the natural log of 2 (ln 2) by the terminal elimination rate constant (λz). The maximum plasma concentration (C_max_) and the time to maximum concentration for Mg^2+^ were directly determined from the plasma concentration of Mg^2+^. The area under the curve (AUClast) was calculated using the log-linear trapezoidal rule.

### Statistical Analysis

Data were analyzed across time using a commercially available software (R 3.5 Statistical Software). Raw data were tested for normality using Shapiro-Wilk test and graphed using SigmaPlot. The paired *t*-test was used to analyze for significance for each ECG and blood pressure variable, and the electrolyte concentrations across the two treatment groups. A linear mixed effects model was used to determine significance across time points. For observational behavior data, ANOVA, poisson, and logistic models were fit based on the observations for head elevation (treated as a likert scale), ear movement (count data), and action (binary). Each of these responses were fitted using a repeated measures design, since each horse was recorded over time. Significance was set at *P* < 0.05.

## Results

### Plasma Analysis

**Figure 4:**
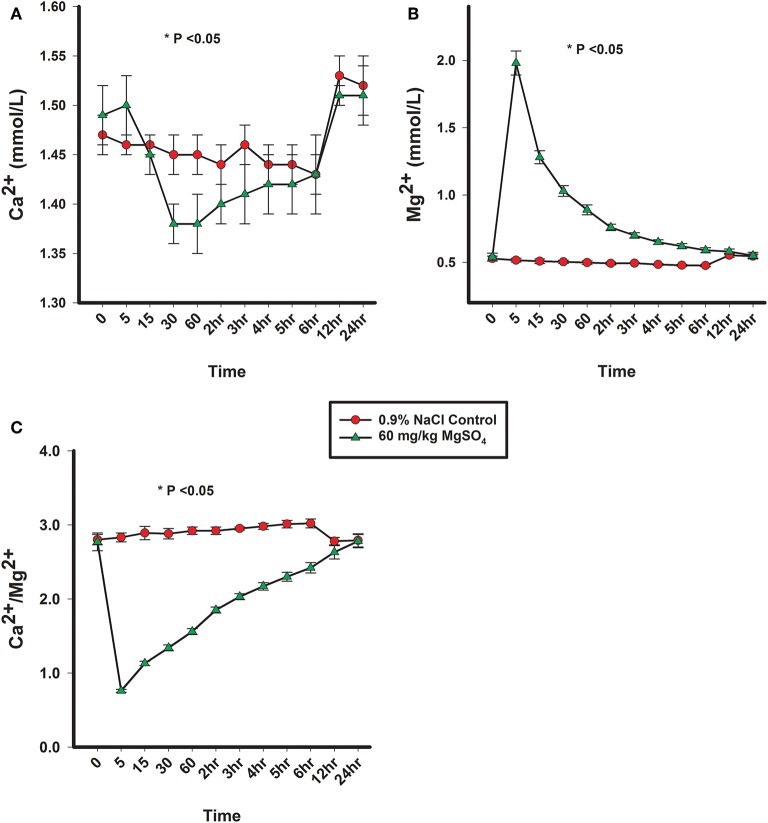
**(A)** Plasma Ca^2+^ concentration, **(B)** plasma Mg^2+^ concentration, and **(C)** ratio of plasma Ca^2+^ to plasma Mg^2+^ concentrations from horses administered intravenously 60 mg/kg MgSO_4_ (green triangles) and equivalent volume of 0.9% NaCl control horses (red circles). Results are presented as mean ± SE (^*^P < 0.05).

There was a significant difference of treatment between the control group and the MgSO_4_ group or Ca^2+^, Mg^2+^, and Ca^2+^/Mg^2+^. The plasma concentration of Mg^2+^ increased rapidly following the administration of MgSO_4_ and remained significantly elevated from 5 min until 2 h; plasma Mg^2+^ did not return to baseline levels until 24 h. Plasma Ca^2+^ decreased by 30 min and remained lower until the 3rd hour (*P* < 0.001) as compared to the baseline concentration, The ratio of Ca^2+^ to Mg^2+^ declined immediately from a baseline ratio of 2.77 ± 0.12 SE to a ratio of 0.76 ± 0.0.02 SE at 5 min and then gradually increased, but remained significantly lower than baseline until 5 h (*P* < 0.05) (Figures 4A–C, Table 1).

**Table 1:** Plasma electrolytes in five horses administered 60 mg/kg of MgSO_4_ and equivalent volume of 0.9% NaCl.

	**Baseline**	**5 Min**	**15 Min**	**30 Min**	**60 Min**	**2 H**	**3 H**	**4 H**	**5 H**	**6 H**	**12 H**	**24 H**
**0.9% NaCl Control**												
pH (H^+^)	7.3 ± 0.05	7.31 ± 0.03	7.3 ± 0.04	7.28 ± 0.04	7.3 ± 0.03	7.31 ± 0.02	7.31 ± 0.03	7.31 ± 0.04	7.32 ± 0.03	7.32 ± 0.03	7.28 ± 0.03	7.27 ± 0.02
Ca^2+^ (mmol/L)	1.47 ± 0.02	1.46 ± 0.01	1.46 ± 0.01	1.45 ± 0.02	1.45 ± 0.02	1.44 ± 0.02	1.46 ± 0.02	1.44 ± 0.02	1.44 ± 0.02	1.43 ± 0.02	1.53 ± 0.02	1.52 ± 0.03
Mg^2+^ (mmol/L)	0.53 ± 0.02	0.52 ± 0.01	0.51 ± 0.02	0.5 ± 0.01	0.5 ± 0.01	0.49 ± 0.01	0.49 ± 0	0.48 ± 0.01	0.48 ± 0.01	0.48 ± 0.01	0.55 ± 0.01	0.55 ± 0.01
TCO_2_ (mmol/L)	29.68 ± 0.62	28.18 ± 0.48	27.84 ± 0.88	28.84 ± 0.69	28.94 ± 0.59	28.92 ± 0.37	28.82 ± 0.44	28.16 ± 0.58	27.92 ± 0.31	28.68 ± 0.2	26.48 ± 0.6	27.52 ± 0.76
Ca^2+^/Mg^2+^ (mol/mol)	2.8 ± 0.07	2.83 ± 0.06	2.89 ± 0.09	2.88 ± 0.07	2.92 ± 0.05	2.92 ± 0.05	2.95 ± 0.02	2.98 ± 0.04	3.01 ± 0.05	3.02 ± 0.06	2.78 ± 0.05	2.79 ± 0.09
**60 mg/kg MgSO**_**4**_												
pH (H^+^)	7.28 ± 0.02	7.3 ± 0.02	7.28 ± 0.03	7.28 ± 0.02	7.28 ± 0.02	7.28 ± 0.02	7.3 ± 0.03	7.31 ± 0.04	7.33 ± 0.03	7.32 ± 0.03	7.31 ± 0.02	7.32 ± 0.02
Ca^2+^ (mmol/L)	1.49 ± 0.03	1.5 ± 0.03	1.45 ± 0.02	1.38 ± 0.02^*^	1.38 ± 0.03^*^	1.4 ± 0.02^*^	1.41 ± 0.03^*^	1.42 ± 0.03^*^	1.42 ± 0.03^*^	1.43 ± 0.04^†^	1.51 ± 0.01	1.51 ± 0.03
Mg^2+^ (mmol/L)	0.54 ± 0.03	1.98 ± 0.09^*^	1.28 ± 0.05^*^	1.03 ± 0.04^*^	0.89 ± 0.04^*^	0.76 ± 0.02^*^	0.70 ± 0.02^*^	0.65 ± 0.02	0.62 ± 0.02	0.59 ± 0.02	0.58 ± 0.02	0.55 ± 0.02
TCO_2_ (mmol/L)	28.48 ± 1.04	26.58 ± 0.7^*^	27.42 ± 0.69^*^	27.98 ± 0.61	28.72 ± 0.58	28.38 ± 0.4	28.22 ± 0.64	28.68 ± 0.63	27.64 ± 0.57^*^	28.48 ± 0.37	27.78 ± 1.1^*^	27.22 ± 0.73^*^
Ca^2+^/Mg^2+^ (mol/mol)	2.77 ± 0.12	0.76 ± 0.02^*^	1.13 ± 0.03^*^	1.34 ± 0.04^*^	1.56 ± 0.04^*^	1.85 ± 0.04^*^	2.03 ± 0.04^*^	2.17 ± 0.05^*^	2.3 ± 0.06^*^	2.42 ± 0.07	2.63 ± 0.09	2.78 ± 0.09

*Values presented as mean ± SE*.

* *Significant (P ≤ 0.05)*.

† *Near significance (P ≤ 0.07)*.

### Cardiovascular

#### ECG

**Figure 5:**
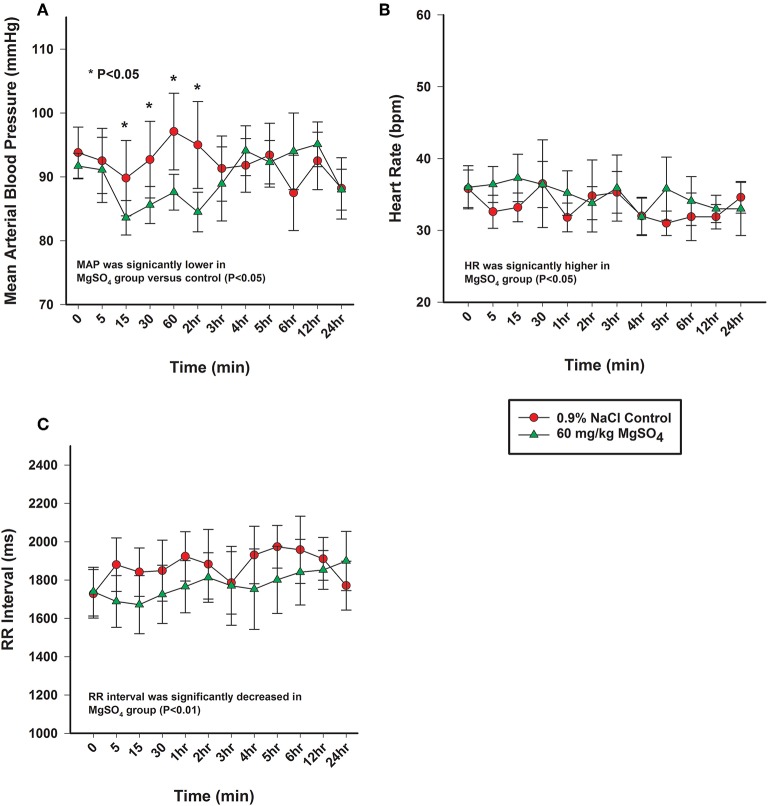
**(A)** Mean arterial pressure (mmHg), **(B)** heart rate (bpm), and **(C)** electrocardiographic RR interval (ms) from horses administered intravenously 60 mg/kg MgSO_4_ (green triangles) and equivalent volume of 0.9% NaCl (red circles). Results are presented as mean ± SE (^*^P < 0.05).

**Figure 6:**
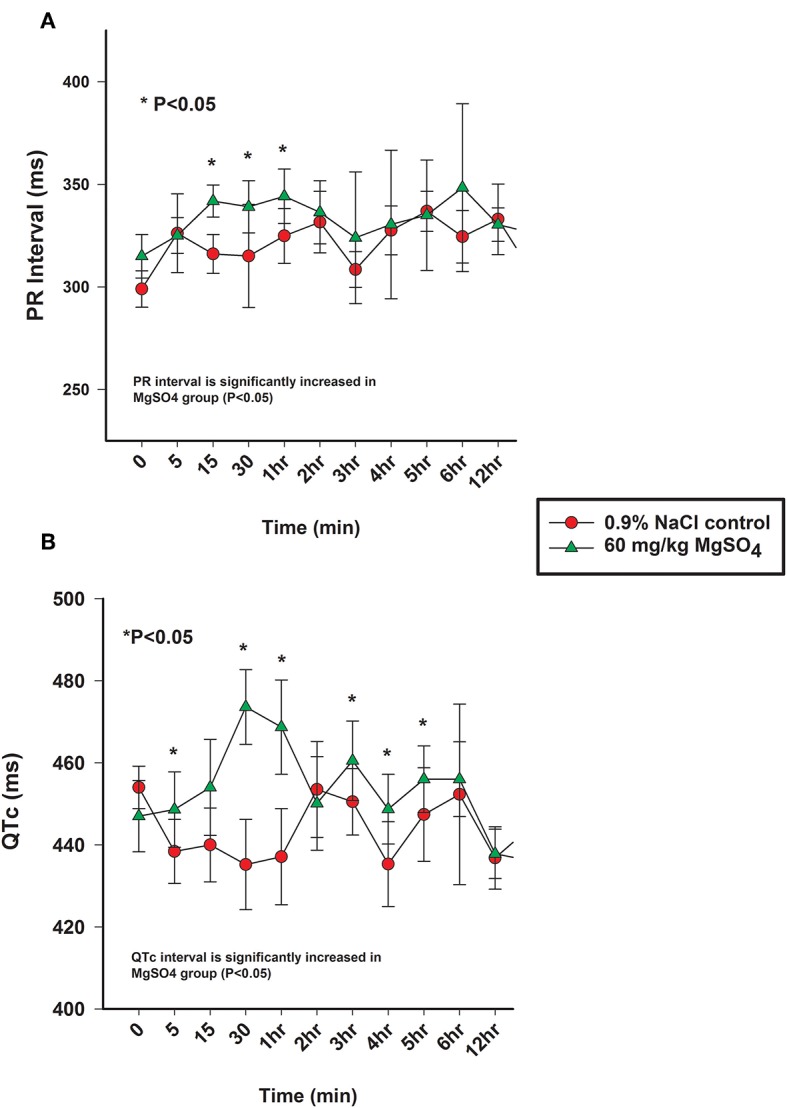
**(A)** PR interval (ms) and **(B)** QTc intervals (ms) from the analysis of electrocardiograms from horses administered intravenously 60 mg/kg MgSO_4_ (green triangles) and equivalent volume of 0.9% NaCl (red circles). Results are presented as mean ± SE (^*^P <0.05).

The heart rate increased significantly with MgSO_4_ administration (*P* < 0.05). As compared to baseline in the MgSO_4_ group, the HR increased by 5 min and remained higher than the control group until 30 min (Figure 5B). Intervals for RR, PR, and QTc differed between groups (*P* < 0.05). The RR interval decreased from a baseline value of 1,739 ± 127.4–1,671 ± 151.4 ms by 15 min and returned to baseline values by 60 min (Figure 5C). The PR interval significantly increased over the first 15 min and remained significantly increased through 2 h, and was significant between groups (Figure 6A, Table 2). The QTc interval was significantly increased in the MgSO_4_ treatment group as compared to the control group. As compared to baseline, the QTc interval was longer at 30 min, 1, 3, 4, 5, 6 h time points (*P* < 0.05), and at the 2 h time point, was nearly significant (*P* < 0.055) compared to the control group (Figure 6B).

**Table 2:** ECG intervals in five horses administered 60 mg/kg of MgSO_4_ and equivalent volume of 0.9% NaCl.

	**Baseline**	**5 Min**	**15 Min**	**30 Min**	**60 Min**	**2 H**	**3 H**	**4 H**	**5 H**	**6 H**	**12 H**	**24 H**
**0.9% NaCl Control**												
HR (bpm)	36 ± 2.6	33 ± 2.3	33 ± 2.0	36 ± 6.1	32 ± 2.0	35 ± 5.0	35 ± 2.9	32 ± 2.6	31 ± 1.7	32 ± 3.3	32 ± 1.7	35 ± 2.2
PR (ms)	299 ± 8.9	326 ± 19.2	316 ± 9.5	315 ± 25.1	325 ± 13.4	332 ± 15.0	309 ± 8.7	328 ± 11.9	337 ± 9.8	324 ± 12.8	333 ± 17.2	306 ± 7.9
QRS (ms)	136 ± 5.2	138 ± 4.8	134 ± 4.4	133 ± 4.9	136 ± 3.8	140 ± 5.3	138 ± 5.1	139 ± 6.1	141 ± 6.0	141 ± 6.8	137 ± 5.2	136 ± 4.4
RR (ms)	*1, 728*±126.7	*1, 880*±139.7	*1, 841*±126.3	*1, 849*±159.2	*1, 923*±128.9	*1, 882*±181.3	*1, 785*±163.1	*1, 930*±149.9	*1, 973*±111.5	*1, 958*±175.2	*1, 910*±111.5	*1, 771*±127.7
QT (ms)	590 ± 21.0	598 ± 24.1	595 ± 23.4	586 ± 41.2	604 ± 27.7	613 ± 26.5	593 ± 25.6	602 ± 25.9	624 ± 20.4	625 ± 17.6	602 ± 18.9	586 ± 21.2
QTcB (ms)	454 ± 5.2	438 ± 7.8	440 ± 9.0	435 ± 11.0	437 ± 11.7	454 ± 11.7	450 ± 8.1	435 ± 10.3	447 ± 11.4	452 ± 22.0	437 ± 7.6	444 ± 12.5
**60 mg/kg MgSO**_**4**_												
HR (bpm)	36 ± 3.0	36 ± 2.5	37 ± 3.3	36 ± 3.2	35 ± 3.1	34 ± 2.3^*^	36 ± 4.6	40 ± 7.9	36 ± 4.4	34 ± 3.4	33 ± 1.9	33 ± 3.7
PR (ms)	315 ± 10.6	325 ± 8.7	342 ± 7.8^*^	339 ± 12.7^*^	344 ± 13.3^*^	336 ± 15.4^*^	324 ± 32.1	330 ± 36.2	335 ± 26.9	348 ± 40.9	330 ± 8.2	326 ± 13.3
QRS (ms)	143 ± 4.7	143 ± 4.2	142 ± 5.1	144 ± 5.5	142 ± 2.9	144 ± 6.0	143 ± 6.0	143 ± 2.9	145 ± 5.6^†^	143 ± 7.4	140 ± 3.5	144 ± 4.0
RR (ms)	*1, 739*±127.4	*1, 688*±135.2	*1, 671*±151.4	*1, 725*±152.1	*1, 765*±136.3	*1, 813*±128.9^*^	*1, 770*±206.1	*1, 752*±210.2	*1, 801*±175.3	*1, 841*±171.5	*1, 852*±101.3	*1, 899*±154.3
QT (ms)	581 ± 22.0	579 ± 17.2	581 ± 26.6	615 ± 27.5	618 ± 23.3^*^	604 ± 28.3	608 ± 45.1	587 ± 46.8	604 ± 32.9	613 ± 28.7	592 ± 13.9	593 ± 24.4
QTcB (ms)	447 ± 8.7	449 ± 9.2	454 ± 11.7	474 ± 9.1^*^	469 ± 11.5^*^	450 ± 11.4^†^	460 ± 9.7^*^	449 ± 8.5^*^	456 ± 8.1^*^	456 ± 9.1^*^	438 ± 6.0	436 ± 20.6

*Values presented as mean ± SE*.

* *Significant (P ≤ 0.05)*.

† *Near significance (P ≤ 0.07)*.

#### Blood Pressure

The mean arterial blood pressure (MAP) decreased from 92 ± 2.0 to 84 ± 2.7 mmHg within the first 15 min after administration of MgSO_4_, not seen with 0.9% saline, and slowly returned to baseline levels by 3 h (Figure 5A). MAP differed and was lower than control (*P* < 0.05), and was lower than baseline from 15 min to the 2 h time period (*P* = 0.029). Both systolic arterial blood pressure (SBP) and diastolic arterial blood pressure (DBP) differed between the two treatment groups (*P* < 0.05). SBP decreased from baseline from 15 min to the 2 h time period (*P* < 0.05) and DBP decreased from baseline at 15, 30 min and 2 h (*P* < 0.05) (Table 3) after MgSO_4_ administration.

**Table 3:** Blood pressure variables for five horses administered 60 mg/kg MgSO_4_ and equal volume of 0.9% NaCl (control).

	**Baseline**	**5 Min**	**15 Min**	**30 Min**	**60 Min**	**2 H**	**3 H**	**4 H**	**5 H**	**6 H**	**12 H**	**24 H**
**0.9% NaCl Control Group**												
Beat to beat (ms)	1,714±119.7	1,879±138.5	1,841±128.1	1,849±163.5	1,929±128.8	1,925±148.5	1,787±159.0	1,925±149.9	1,967±110.5	1,928±200.7	1,911±110.3	1,768±126.4
HR (bpm)	37 ± 2.8	33 ± 2.3	33 ± 2.1	37 ± 6.7	32 ± 2.1	32 ± 2.5	36 ± 3.0	32 ± 2.7	31 ± 1.7	33 ± 4.4	32 ± 1.6	35 ± 2.3
dp/dt+ (mmHg/s)	597 ± 132.4	569 ± 144.1	461 ± 54.7	454 ± 55.0	455 ± 47.7	414 ± 76.6	475 ± 60.4	436 ± 70.4	413 ± 57.3	711 ± 311.9	475 ± 53.1	514 ± 58.4
SBP (mmHg)	118 ± 5.9	116 ± 7.1	112 ± 8.2	115 ± 6.9	121 ± 7.5	119 ± 9.0	116 ± 6.2	116 ± 6.3	118 ± 6.9	110 ± 7.4	116 ± 6.3	112 ± 5.8
DBP (mmHg)	75 ± 3.5	74 ± 5.1	71 ± 5.7	75 ± 6.1	79 ± 5.9	77 ± 7.0	73 ± 5.5	74 ± 4.0	77 ± 4.7	71 ± 6.4	75 ± 4.5	69 ± 5.3
MBP (mmHg)	94 ± 4.0	92 ± 5.1	90 ± 5.9	93 ± 6.0	97 ± 6.0	95 ± 6.8	91 ± 5.1	92 ± 4.2	93 ± 5.0	88 ± 5.9	93 ± 4.5	88 ± 4.8
Pulse Pressure (mmHg)	42 ± 3.3	42 ± 3.8	41 ± 3.5	39 ± 2.4	42 ± 3.5	42 ± 3.8	42 ± 3.1	42 ± 3.7	41 ± 3.1	40 ± 2.9	41 ± 2.3	43 ± 1.3
D_DN time	893 ± 44.1	824 ± 29.4	868 ± 66.6	840 ± 49.9	889 ± 42.3	842 ± 53.3	878 ± 56.7	835 ± 44.1	945 ± 54.8	841 ± 49.5	823 ± 66.0	852 ± 60.3
Area under the curve from diastole to diastole	34 ± 3.5	36 ± 3.5	33 ± 3.2	32 ± 3.4	34 ± 2.9	35 ± 3.7	33 ± 3.1	34 ± 3.5	35 ± 2.9	35 ± 4.8	33 ± 2.5	32 ± 2.2
Pressure at dicrotic notch (mmHg)	94 ± 2.3	96 ± 3.6	92 ± 3.9	96 ± 4.5	99 ± 4.3	99 ± 6.6	92 ± 4.1	95 ± 4.1	96 ± 4.0	92 ± 6.2	96 ± 3.8	89 ± 4.8
rate × pressure	4 ± 0.4	4 ± 0.5	4 ± 0.5	4 ± 1.0	4 ± 0.5	4 ± 0.4	4 ± 0.5	4 ± 0.4	4 ± 0.4	4 ± 0.3	4 ± 0.4	4 ± 0.4
**60 mg/kg MgSO**_**4**_												
Beat to beat (ms)	1,738±130.2	1,685±134.3	1,671±152.6	1,728±151.2	1,780±123.6	1,816±128.3	1,769±205.5	1,739±219.8	1,797±177.3	1,825±182.8	1,855±99.4	1,899±153.4
HR (bpm)	36 ± 3.2	37 ± 2.5	38 ± 3.4	37 ± 3.4	35 ± 2.7	34 ± 2.4	36 ± 5.0	41 ± 8.9	36 ± 4.5	35 ± 4.1	33 ± 2.0	33 ± 3.8
dp/dt+ (mmHg/s)	523 ± 84.1	588 ± 50.2	579 ± 78.1	503 ± 58.5	455 ± 59.6	489 ± 96.5	438 ± 53.8	653 ± 267.8	471 ± 51.7	546 ± 132.3	463 ± 66.1	461 ± 82.3
SBP (mmHg)	116 ± 3.0	116 ± 6.4	107 ± 4.1^*^	108 ± 4.8^*^	110 ± 4.2^*^	107 ± 5.0^*^	111 ± 8.4	118 ± 6.1	115 ± 5.6	116 ± 8.0	118 ± 5.0	111 ± 4.2^*^
DBP (mmHg)	73 ± 2.8	71 ± 4.9	65 ± 2.9^*^	68 ± 3.2^*^	71 ± 3.3	68 ± 3.4^*^	72 ± 5.8	76 ± 4.1	74 ± 4.0	76 ± 6.2	78 ± 3.6	71 ± 4.1
MBP (mmHg)	92 ± 2.0	91 ± 5.1	84 ± 2.7^*^	86 ± 2.9^*^	88 ± 2.8^*^	85 ± 3.1^*^	89 ± 5.8	94 ± 3.9	92 ± 3.4	94 ± 6.0	95 ± 3.5	88 ± 3.2^*^
Pulse Pressure (mmHg)	43 ± 1.8	44 ± 2.9	42 ± 3.0	40 ± 3.2^*^	39 ± 2.6^*^	39 ± 3.3^*^	39 ± 4.4	42 ± 4.7	40 ± 3.3	40 ± 3.3	40 ± 2.5	40 ± 2.3
D_DN time	889 ± 79.4	882 ± 64.3	811 ± 33.9	755 ± 33.6	782 ± 40.0	819 ± 34.7	812 ± 44.6	839 ± 74.7	829 ± 65.6	850 ± 60.4	788 ± 29.7	828 ± 61.1
Area under the curve from diastole to diastole	34 ± 2.3	33 ± 2.7	31 ± 2.2^*^	30 ± 2.5^*^	30 ± 1.9^*^	30 ± 2.9^*^	30 ± 3.7	34 ± 3.4	38 ± 3.4	31 ± 3.0	32 ± 2.5	33 ± 3.2
Pressure at dicrotic notch (mmHg)	93 ± 3.4	91 ± 3.4	85 ± 2.0	88 ± 2.0	90 ± 2.3	87 ± 2.6	90 ± 4.2	97 ± 4.6	95 ± 3.3	97 ± 6.4	99 ± 2.7	92 ± 2.9
rate × pressure	4 ± 0.3	4 ± 0.5	4 ± 0.5	4 ± 0.5	4 ± 0.4	4 ± 0.3	4 ± 0.8	5 ± 1.3	4 ± 0.6	4 ± 0.4	4 ± 0.4	4 ± 0.3

*Values represented as mean ±SE*.

* *Significant (P ≤ 0.05)*.

*^†^Near significance (P ≤ 0.07)*.

### Locomotion

**Figure 7:**
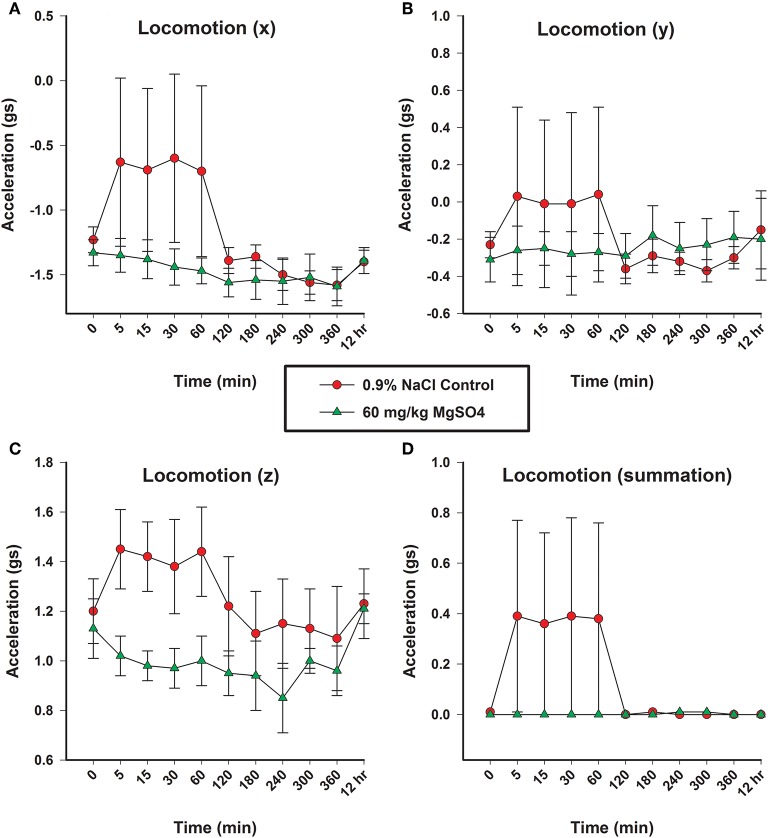
Locomotion as measured in three planes (x, y, and z) by acceleration (gs), using accelorometers housed in the base transmitter located on the withers; **(A)** x-vector plane, **(B)** y-vector plane, **(C)** z-plane, and **(D)** overall locomotion. Horses received MgSO_4_ intravenously (green triangles) or an equivalent volume of 0.9% NaCl (red circles).

There was a significant decrease in activity in x, y and z-axis and in overall acceleration axes for the MgSO_4_ treatment group compared to the control group (Figures 7A–D).

### Observational Behavior Data

Ear movement was significantly less, 4.2 ± 0.65 movements over 30 s, in the MgSO_4_ treatment group compared to 5.8 ± 1.55 ear movements over 30 s in the control group (*P* < 0.01).

### Pharmacokinetic

The selected reported pharmacokinetic parameters were calculated with the endogenous baseline values for Mg^2+^ subtracted prior to analysis. This normalization was done for each horse at each time point within the MgSO_4_ treatment group. Non-compartmental analysis provided a maximum concentration (C_max_) of 1.44 ± 0.19 mmol/L and an average time of maximal observed concentration (T_max_) of 5.0 min. The average clearance (CL) was 164.7 ± 62.7 L/min and the observed average volume of distribution (Vss_obs) was 1.2 ± 0.38 L/kg.

### Temperature

There was no significant change from baseline in either group or across time (36.4 ± 0.2°C).

### Instrumentation

All instrumentation functioned properly for the duration of the study, and no horses experienced any complications from the implanted catheters. One issue that occurred was that the ECG electrodes would occasionally detach from the horse. Electrodes were reaffixed as necessary, but there were no lapses in data collection. Horses appeared to be undisturbed by the placement of the collar.

## Discussion

The most important findings of our study was that the bolus IV administration of MgSO_4_ resulted in changes in electrophysiology of the heart, reduced blood pressure, and reduced locomotion, and these changes may be responsible for the calming behavior in horses. This is the first report documenting these pharmacodynamic effects which may be directly related to the inappropriate use of MgSO_4_ in horses.

The direct arterial blood pressure is the most accurate means for detecting changes in the cardiovascular tone of mammals (15). Our study confirmed, with appropriate controls, that mean arterial blood pressure decreased in an amount similar to reported values for beta-adrenergic antagonists that are also clinically effective to not only reduce blood pressure, and in humans can cause fatigue (16). In a review of 56 randomized controlled trials examining the blood pressure lowering effects of beta-1 selective blockers, the average reduction in blood pressure was 10 and 8 mmHg in systolic and diastolic blood pressure, respectively, in patients with mild to moderate hypertension (17). An associated side effect of the beta-blocker propranolol is fatigue (18). In our study, the observed changes in systolic and diastolic blood pressures were 9 and 8 mmHg, respectively, at the 15 min time point, which is similar to the reports in humans treated with beta-1 selective blockers. This change in blood pressure upon administration of MgSO_4_ could be causing a similar sensation of fatigue, possibly noted as calming, in horses.

The use of telemetric monitoring to detect physiologic changes is not a novel approach in animal research, this method has been used to measure changes in hemodynamic parameters and physiologic responses to drug administrations in other species (19–22). A previous study examined the wireless invasive blood pressure monitoring in ponies, but this study did not use the implanted devices for more than 48 h (23). To our knowledge, ours is the first study to implant horses with invasive chronic telemetric catheters to serially measure changes in arterial blood pressure, core temperature, locomotion, and ECG changes in response to drug administration. This system allowed for the direct monitoring of the desired variables in horses while allowing them freedom of movement to assess their activity. The use of this invasive technology limited the number of test subjects as it was not practical to implant a larger number of horses, but the increased accuracy and decreased variability associated with handling, reduced the number of animals needed to show significant effects. This method appeared to be reliable, accurate, and most importantly, successful with the horse being allowed free movement. This novel method could be used in future studies to evaluate the effect of drugs on the cardiovascular physiologic variable and locomotion. The unit also can be used without the implanted telemetric catheters to gather only ECG and locomotion data.

In previous work by these authors, changes in blood pressure and heart rate could not be compared to a control group because of the difficulty in restraining instrumented horses without the benefit of the test article (**?**). The data in the current study, as to be expected, was similar to prior data, but in this study it was possible to utilize a control group which received an equivalent volume of saline. It is not thought that the lack of a control group affected prior data, but now the effect has been confirmed. The use of a control group allowed for analysis of the effect of treatment which identified significant changes in HR and MAP, and ECG intervals for PR, QRS, and QTc. These findings are consistent with reported results following MgSO_4_ administration to a variety of species (24, 25). Following the administration of MgSO_4_ the decrease in MAP was less significant, but the pressure waveforms were acquired from a more central artery than the previously used facial artery. There are inherent differences in the placement of the catheter for monitoring with respect to the size of the artery vs. the size of the catheter tip, as well as, the height of the monitoring position above the heart, and the use of fluid filled catheters vs. get tips. However, as Mg^2+^ has been shown to lower blood pressure (2, 26, 27), and act as a peripheral vasodilator by serving as a Ca^2+^ antagonist in vascular smooth muscle (28–30), and has been shown to effect vasodilation of coronary vessels (2, 31, 32), these changes were not unexpected. Little is known about its effect on larger arterial vessels, but a direct effect has been demonstrated on the peripheral vasculature where Mg^2+^ has been shown to lower arterial pressure by causing significant vasodilation of intact arterioles and venules (29). There is an important association between blood pressure and heart rate, as MAP declines, a baroreceptor- mediated reflexive increase in HR to maintain cardiac output is the usual result (33). As opposed to the administration of beta 1-selective blockers, with the administration of MgSO_4_, there was no decrease in heart rate, but rather an increase in heart rate; which is consistent with a baroreceptor mediated reflex. There was an increase in heart rate at the 5 min time point and nearly significant increase in heart rate at the 15 min time point, and an increase in heart rate across treatment, but not as significant as anticipated. This diminished effect on heart rate could be due to the fact MgSO_4_ has also been shown to have a sympatholytic effect by inhibiting norepinephrine release (34), and this mechanism could also be contributing to decreasing blood pressure independent of peripheral vasodilation.

While heart rate was mildly increased, there were very relevant changes identified with the ECG intervals. It's important to note that the ECG interval analysis was conducted using a very sensitive analysis program, capable of more sensitive analysis of interval duration than by visual observation or manual measurement using an ECG ruler. This software is capable of measuring to the thousands of a second as compared to the tenths of a second common with manual measurement. When viewing the ECG waveforms in real time, no differences were noticeable to any of the investigators. It was only through the high resolution and sensitivity of the ECG analysis program that these significant interval conduction time changes were detectable. These ECG intervals represent the conduction time of electrical impulses across the atria, through the atrioventricular node (AV) and the bundle of His, and across the ventricles. Changes to these intervals can be representative of a delay in the transmission of the electrical impulses through the heart.

The shortening of the RR interval was statistically different between MgSO_4_ and 0.9% saline significant across treatment and for multiple early time points (*P* < 0.01). The RR interval might be a more sensitive indicator of HR changes as it measures time from beat to beat as opposed to number of beats, which could be less sensitive due to the low heart rate of horses. While The RR interval decreased following MgSO_4_ administration, the PR and QTc intervals increased. In similar studies involving MgSO_4_, increased PR and QRS intervals were detected (35–38). The PR interval in the MgSO_4_ treatment group increased from a baseline of 315 ± 10.6–344 ± 13.3 ms by 60 min, and reflected a change in the atrioventricular nodal conduction time (37). It has been proposed that the mechanism of action for altering the conduction times could be direct, such as the blockage of calcium channels (30), which is time and voltage dependent (32), or as an indirect mechanism of altering peripheral vascular resistances and autonomic tone. The alteration of electrical conductance in the heart explains why MgSO_4_ has been used as an antiarrhythmic in humans to treat torsade de pointes (39, 40) and other ventricular arrhythmias (41, 42). MgSO_4_ possesses many properties that effect the electrophysiological functioning of the heart, but potentially the most relevant antiarrhythmic activity is the inhibition of calcium channels (43, 44). QT intervals typically shorten during tachycardia and extend during bradycardia, and represents ventricular depolarization and repolarization (45). HR can create variability in measuring the QT interval; it is necessary to correct for HR, and in this study, Bazett's formula was utilized to generate a QT corrected value (QTc). The prolongation of the QTc in the MgSO_4_ treatment group was most likely due to Mg^2+^ blocking the influx of Ca^2+^ into the ventricular cardiomyocytes and extending the rapid depolarization phase. As previously, stated similar studies in other species identified increases in PR and QRS intervals following MgSO_4_ administration, with no increase in QTc intervals. In our study, the QTc interval was significantly increased in the MgSO_4_ treatment group from a baseline of 447 ± 8.7–474 ± 9.1 ms then returned to baseline values by 60 min. While prolongation of the QTc can be congenital or acquired through drug induction, there were no visible ventricular arrhythmias or visual changes in duration that could be considered as dangerous or as a prelude to ventricular tachycardia or fibrillation. It has been reported from the field that some horses have collapsed and even succumbed to intravenous administration of MgSO_4_ following very rapid infusions; for this reason, the 5 min duration of infusion was used for this study. However, it is possible that the changes in ECG intervals for a 5 min infusion could be even more pronounced and potentially fatal with a very rapid infusion, and account for the reports from the field.

As total magnesium is not active, only Mg^2+^ was monitored. The changes in Mg^2+^ and Ca^2+^ observed in this experiment were expected and consistent with previous research documenting experimentally induced hypermagnesemia (13, 14). As a result of the administration of MgSO_4_ in this study, there were obvious and expected changes in the plasma concentration of Mg^2+^, Ca^2+^, and the ratio of Ca^2+^ to Mg^2+^, some cardiovascular variables, as well as, changes in activity and observed behavior. The increase in Mg^2+^ remained significantly elevated for 3 h, which allows for its use in regulatory control. The decrease in Ca^2+^ persisted for 5 h and the ratio of Ca^2+^ to Mg^2+^ remained depressed for 5 h. The increase in Mg^2+^ and decrease in Ca^2+^ both obviously contributed to the change in their ratio. In reported literature, the decrease in Ca^2+^ following MgSO_4_ was not as significant as reported here, or if it was, did not decline for this extended period of time. One explanation is a lower dose of MgSO_4_ was administered (40 mg/kg) (13). In dogs, the changes associated with concentrations of Mg^2+^ and Ca^2+^ have been found to be dependent upon the dose of MgSO_4_ administered as a continuous infusion or as a bolus (24).

The MgSO_4_ treatment group of horses demonstrated less activity with little variability, while the control group had increased activity coinciding with the end of the 0.9% NaCl injection. It is important to acknowledge that the increase in activity for the control group occurred immediately after the horses were turned loose following the 5 min administration. Members of the research team who were blinded to treatment, described the movement of the control group as “searching” or “exploring” in the context of a novel environment. Additionally, the control group of horses had a great deal of variability over the first hour of the experiment. The goal in observing the accelerometers was not to analyze differences between the separate planes, but to evaluate overall activity. Following the introduction to the novel environment, the activity level for the control group decreased around 2 h. Beyond the 2 h time point, the activity levels between the two groups was very similar, so it was difficult to differentiate between the two groups. This timing is consistent with the desired effect competitors are looking for; 2 h following the administration of MgSO_4_ the locomotion of the two treatment groups was not different.

The behavior of the horses was observed by research team members blinded to treatment. Of the three subjectively recorded observation categories, ear movement was the only one statistically significant with the control group having a greater number of ear movements over a 30 s observation period. Horses prick their ears when curious or exploring, and lie quiet with minimal movement while relaxed or resting. Ear movement is commonly evaluated to determine sedation and arousal in horses. While the research team was blinded to treatment group, it became obvious to them which horses had received MgSO_4_. In humans, the intravenous administration of 6 g of 4% MgSO_4_ (over 6 min) resulted in patients reporting a sensation of increased warmth with flushing and sweating (46). These clinical signs were associated with a transient decrease in blood pressure (46). In our study, three of the five horses administered MgSO_4_, were observed to exhibit a combination of licking and chewing behavior during the administration or immediately following the conclusion of the administration.

The use of a novel telemetric approach for the acquisition of physiologic signals had an impact on the analysis of the pharmacokinetics of Mg^2+^ following the administration of MgSO_4_. In an attempt to limit human-horse interactions, fewer time points were assigned for blood collection. As a result, there were fewer data points available from the semi-log concentration-time plot. It is typical to fit at least three of the final data points to a linear regression; more is better if possible to maximize r2. For this study the geometric mean of data points used for the determination of the terminal elimination rate constant (Lambda_z; K_el_) was 4.2 with a minimum of 3 and a maximum of 6. The K_el_ reported in this study was the geometric mean of 0.187 mmol/h with a minimum of 0.119 and maximum of 0.29. This value is slightly higher than the 0.13 mmol/h reported in previous work by these authors (submitted paper) and can be explained by the reduction in terminal data points. The terminal half-life of the terminal phase (HL_Lambda z; *t*_1/2_) reported in this study was the geometric mean of 3.7 h with a minimum of 2.4 and maximum of 5.8 h, and previously reported values with the same administration strategy, but different collection time points, was 5.5 ± 0.65 SD hours. In humans the terminal half-life for intravenous Mg^2+^ has been reported to be around 3 h (47, 48). As *t*_1/2_ is dependent upon the clearance of the drug and the volume of distribution, the increased Cl observed for this study can account for part of the decrease in *t*_1/2_; as clearance increases, there is less “drug” in the plasma at each of the successive time points. With fewer time points available, the AUClast is impacted and can represent an underestimation of the true value when using the log-linear trapezoidal method. As AUC and Cl are inversely related, a lower AUC can provide a higher Cl and therefore a lower *t*_1/2_. Overall, the pharmacokinetic values reported for a single intravenous bolus of 60 mg/kg MgSO_4_ are similar to previous work in horses.

## Conclusion

This study identified a decrease in blood pressure as a result of a 60 mg/kg intravenous bolus of MgSO_4_. Concurrently, increases in HR were most likely due the baroreceptor reflex. The changes in PR and QTc intervals were most likely due to a direct effect of the plasma Mg^2+^ increase on the reduction of cardiac electrical conduction through the blockade of Ca^2+^ channels. The decrease in locomotor activity and observed behavior were obvious and compelling considering the higher HRs were associated with the horses with the lower locomotor activity; horses in the control group demonstrated an increase in activity with greater variability but had lower HRs, particularly early on after exposure to the new environment.

Horses in this study did not appear to be depressed but rather had a diminished interest in their surroundings. It is likely the similar decrease in blood pressure, following intravenous MgSO_4_ administration, could generate behavior changes or a resistance to the distraction of environmental stimuli as evidenced by the lack of locomotor activity in the MgSO_4_ group. When the control horses were left loose following the administration of the identical volume of 0.9% NaCl, they exhibited a searching or investigatory behavior not observed with the MgSO_4_ treatment group. From a competition standpoint, the ability of horses to ignore environmental distractions of stimuli would provide a competitive advantage. Additionally, the administration of MgSO_4_ provided changes in Mg^2+^ concentration and the ratio of Ca^2+^ to Mg^2+^ that could provide support for regulatory control through the analysis of post competition plasma samples.

Our data supported that intravenous administration of MgSO_4_, can induce a calming effect in the horse, and the potential mechanism could be a decrease in blood pressure, or sympathetic reduction (34), or a combination of mechanisms. In concert with our prior data, demonstrating no increase in Mg^2+^ in the CNS and its similarity in calming seen with other vasodilators, the reduction in blood pressure and sympathetic tone, appear to be the most likely mechanism of calming seen in horses.

## Data Availability Statement

The datasets generated for this study are available on request to the corresponding author.

## Ethics Statement

The animal study was reviewed and approved by The Ohio State University's University Institutional Animal Care and Use Committee and fulfilled ARRIVE guidelines (https://www.nc3rs.org.uk/arrive-guidelines) for the humane use of animals in research.

## Author Contributions

All authors have read and approved the final manuscript. SS contributed to study design, data analysis, sample collection, and preparation of the manuscript. RT contributed to study design and preparation of the manuscript. JL contributed to data analysis and preparation of the manuscript. AB contributed to study design, data analysis, and preparation of the manuscript.

### Conflict of Interest

The authors declare that the research was conducted in the absence of any commercial or financial relationships that could be construed as a potential conflict of interest.

## Footnotes

^1^https://inside.fei.org/sites/default/files/2019-Equine-Prohibited-Substances-List_0.pdf
